# Duration of Type 2 Diabetes Mellitus Alters Orosensory Detection of Sweet and Fat: Insights from a Cross-Sectional Study in a North African Population

**DOI:** 10.3390/nu18030432

**Published:** 2026-01-28

**Authors:** Inchirah Karmous, Hatem Ghouili, Rym Ben Othman, Halil İbrahim Ceylan, Luca Puce, Amira Sayed Khan, Naim Akhtar Khan, Henda Jamoussi, Helmi Ben Saad, Nicola Luigi Bragazzi, Ismail Dergaa

**Affiliations:** 1 Research Unit on Obesity UR18ES01, Faculty of Medicine, University of Tunis El Manar, 15 Rue Jebel Lakhdar, Beb Saadoun, Tunis 1007, Tunisia; inchirah.karmous@yahoo.fr (I.K.); hendajamoussi@gmail.com (H.J.); 2 Centre de Recherche Inserm, U1231 INSERM/UB/Institut Agro, Team—Physiologie de la Nutrition & Toxicologie, Faculté des Sciences de la Vie, Université de Bourgogne Franche-Comté (UBFC), 21000 Dijon, France; amira.khan@u-bourgogne.fr (A.S.K.); naim-akhtar.khan01@u-bourgogne.fr (N.A.K.); 3 Research Unit—Sports Science, Health and Movement, High Institute of Sport and Physical Education of Kef, University of Jendouba, El Kef 8100, Tunisia; hatemghouili@gmail.com; 4 Faculty of Medicine, University of Tunis El Manar, 11 Rue Jebel Lakhdar, Beb Saadoun, Tunis 1007, Tunisia; benothmanr@gmail.com; 5 National Institute of Nutrition and Food Technology of Tunis, 11 Jbal Lakhdar Street, Tunis 1007, Tunisia; 6 Physical Education and Sports Teaching Department, Faculty of Sports Sciences, Atatürk University, 25240 Erzurum, Türkiye; 7 Department of Neuroscience, Rehabilitation, Ophthalmology, Genetics, Maternal and Child Health (DINOGMI), University of Genoa, 16132 Genoa, Italy; luca1puce@gmail.com; 8 Heart Failure (LR12SP09) Research Laboratory, Farhat Hached Hospital, University of Sousse, Sousse 4000, Tunisia; helmi.bensaad@rns.tn; 9 Laboratory for Industrial and Applied Mathematics (LIAM), Department of Mathematics and Statistics, York University, Toronto, ON M3J 1P3, Canada; 10 Higher Institute of Sport and Physical Education of Ksar Saïd, University of Manouba, Manouba 2010, Tunisia; phd.dergaa@gmail.com; 11 Physical Activity Research Unit, Sport and Health (UR18JS01), National Observatory of Sports, Tunis 1003, Tunisia

**Keywords:** body mass index, chemosensory perception, glycemic control, gustatory threshold, linoleic acid, metabolic disease, North Africa, orosensory function, sucrose detection, type 2 diabetes mellitus

## Abstract

**Background:** Gustatory dysfunction represents an underrecognized complication that may influence dietary behaviors and metabolic control. Previous investigations have suggested alterations in taste in patients with diabetes, yet the relationship between disease duration and specific taste modalities remains incompletely characterized. **Aim:** This study aimed to (i) compare orosensory detection thresholds for lipid and sweet tastes between patients with recent type 2 diabetes mellitus (rT2DM) (duration ≤ 5 years) and chronic type 2 diabetes mellitus (cT2DM) (duration > 5 years), and (ii) determine whether diabetes duration is associated with alterations in chemosensory function in a North African population. **Methods:** A cross-sectional comparative pilot study was conducted at the National Institute of Nutrition and Food Technology in Tunis, Tunisia, from April to June 2021. Sixty-seven patients with type 2 diabetes mellitus (T2DM) receiving oral antidiabetic medication were recruited through systematic sampling and divided into two groups: rT2DM (*n* = 30, duration ≤ 5 years) and cT2DM (*n* = 37, duration > 5 years). Orosensory detection thresholds for lipid taste were assessed using eight ascending concentrations of linoleic acid ranging from 0.018 to 12 mmol/L. In contrast, sweet taste thresholds were evaluated using a sucrose concentration series ranging from 0.01 to 5 mmol/L. The three-alternative forced-choice method with an ascending-concentration presentation was employed for both taste modalities. Detection thresholds were defined as the lowest concentration at which participants correctly identified the taste quality. **Results:** Patients with cT2DM exhibited significantly elevated orosensory detection thresholds compared to those with rT2DM for both taste modalities tested. The median linoleic acid detection threshold was 6.000 mmol/L in cT2DM versus 0.058 mmol/L in rT2DM (*p* < 0.001), representing a 107-fold increase in detection threshold. For sweet taste, the median sucrose detection threshold was 1.0 mmol/L in cT2DM compared with 0.5 mmol/L in rT2DM (*p* < 0.001), indicating a 2-fold increase in the threshold. In the overall patient cohort, the duration of diabetes was positively correlated with both fat taste perception thresholds (r = 0.657, *p* < 0.001) and sweet taste perception thresholds (r = 0.466, *p* < 0.001). However, when analyses were performed by diabetes duration-based subgroups, these correlations were observed only for fat taste perception in cT2DM, with no statistically significant correlations found in rT2DM. In multivariate linear regression analyses adjusted for age, body mass index, and sex/gender, the duration of diabetes remained independently associated with fat and sweet taste perception. **Conclusions:** Extended T2DM duration is associated with substantial elevations in orosensory detection thresholds for both lipid and sweet tastes in a North African population. These findings suggest that disease chronicity may contribute to chemosensory impairment, potentially influencing dietary preferences and metabolic control in patients with diabetes.

## 1. Introduction

Diabetes mellitus (DM) has become one of the most significant global health challenges of the contemporary era, affecting approximately 463 million individuals worldwide and representing a leading cause of morbidity and premature mortality [1]. The disease burden extends beyond hyperglycemia to encompass numerous microvascular and macrovascular complications that substantially compromise quality of life and impose considerable economic costs on healthcare systems [2,3]. Within the Middle Eastern and North African (MENA) region, epidemiological surveillance indicates concerning prevalence rates, with Tunisia reporting a type 2 DM (T2DM) prevalence of 12% among adults, positioning this condition as the most frequent metabolic disease in the country [3]. The rising prevalence trajectory observed across the Mediterranean basin has been attributed to complex interactions among genetic predisposition, rapid nutritional transitions characterized by increased consumption of energy-dense foods, declining physical activity levels, and population aging [4,5]. Despite substantial advances in pharmacological management and glucose monitoring technologies, achieving optimal glycemic control remains challenging for many patients, partly due to inadequate adherence to dietary recommendations and suboptimal lifestyle modifications [6,7].

The sensory evaluation of food represents a fundamental determinant of dietary selection, consumption patterns, and, ultimately, metabolic health outcomes in individuals with DM [8,9]. Taste perception is mediated by specialized chemoreceptor proteins localized in gustatory receptor cells of taste buds distributed across the oral cavity, with particular concentration on the tongue surface [4,10]. Classical taste physiology recognizes five primary taste qualities, sweet, salty, bitter, sour, and umami, each mediated by distinct molecular mechanisms involving specific receptor proteins and ion channels [2,4]. Emerging evidence suggests that dietary lipids may constitute a sixth basic taste modality, mediated by specific fatty acid receptors, including cluster of differentiation 36 (CD36) and related proteins [5,11]. The perception of sweet taste is mediated primarily by the heteromeric taste receptor complex, which responds to various sweet-tasting compounds, including sucrose, fructose, and artificial sweeteners [12,13]. Individual variations in taste sensitivity, quantified as detection and recognition thresholds, exhibit considerable heterogeneity across populations and are influenced by multiple factors, including genetic polymorphisms, age, sex/gender, body composition, medication use, and disease states [2,14,15,16,17]. Alterations in gustatory function can profoundly influence food preferences, with individuals exhibiting reduced sensitivity to sweet or fatty tastes potentially consuming greater quantities of sugar-rich or lipid-dense foods to achieve equivalent hedonic satisfaction, thereby contributing to positive energy balance and metabolic dysregulation [11,18,19].

Several lines of evidence suggest that DM may be associated with impaired chemosensory function, although findings remain inconsistent across investigations [6,20,21]. Some studies have reported diminished sweet taste sensitivity among patients with DM compared with healthy controls, potentially contributing to increased preference for sugar-sweetened foods and compromising dietary adherence [9,22,23,24]. Proposed mechanisms underlying diabetic gustatory dysfunction include microangiopathy affecting the lingual blood supply and tongue-tip vascularization, peripheral neuropathy involving gustatory nerve fibers, alterations in taste bud morphology and density, changes in salivary composition, and metabolic derangements that affect intracellular signaling pathways within taste receptor cells [22,25,26,27]. Additional factors implicated include reduced glucagon-like peptide-1 signaling, disordered control of intestinal sweet taste receptors, hyperleptinemia-induced inhibition of gustatory responses, and diabetic neuropathy affecting cranial nerves [28,29,30,31]. However, the specific influence of DM duration on taste perception thresholds remains inadequately characterized. This study was undertaken to fill in this gap of knowledge. More in detail, in the present study, we used a 5-year cutoff to distinguish between chronic and recent T2DM. DM duration of five years or more has commonly been used to indicate a stage at which chronic hyperglycemia-related complications, including peripheral neuropathy and microvascular dysfunction, become more prevalent, both of which are mechanisms potentially involved in taste and other chemosensory impairments [32,33,34].

Most existing investigations have focused on Western or Asian populations, with limited data from MENA cohorts, despite potential regional variation in genetic backgrounds, dietary patterns, and diabetes phenotypes [7,35]. Furthermore, the comparative assessment of multiple taste modalities, particularly the evaluation of both sweet and fat taste perception in relation to disease chronicity, has received insufficient attention in the existing body of scholarly literature.

Based on the identified research gaps regarding the relationship between DM duration and chemosensory function in underrepresented populations [6,7,35], our study aimed to (i) compare orosensory detection thresholds for lipid and sweet tastes between patients with recent T2DM (rT2DM; duration ≤ 5 years) and those with chronic T2DM (cT2DM; duration > 5 years), and (ii) examine whether disease duration is associated with systematic alterations in taste sensitivity within a MENA cohort.

## 2. Materials and Methods

### 2.1. Ethical Approval

This investigation was conducted in accordance with the Declaration of Helsinki [36] and was approved by the Ethical Committee of the National Institute of Nutrition and Food Technology in Tunis, Tunisia (approval number: 03/2021, dated 5 April 2021). All participants provided written informed consent following a comprehensive explanation of study procedures, potential risks, and the voluntary nature of participation. Participants retained the right to withdraw from the study at any time without consequence to their medical care.

### 2.2. Sample Size Calculation

Based on our pilot data, a sample size calculation was performed to compare taste detection thresholds between patients with rT2DM and cT2DM.

Since the outcome is continuous (taste detection threshold), the standard formula for two independent means applies:

$$n=\frac{2\cdot {\left(Z_{\alpha /2}+Z_{\beta }\right)}^{2}\cdot \sigma^{2}}{{∆}^{2}}$$

where

Δ = expected mean difference between rT2DM and cT2DM

σ = pooled standard deviation

α = 0.05 (two-sided)

Power = 0.8 (or 80%)

Using the observed difference in detection thresholds for sweet taste (β = 0.61) [9] and assuming a standard deviation comparable to the observed effect size, we estimated that approximately 35 participants per group would provide 80% power to detect a statistically significant difference at the 5% significance level.

### 2.3. Population and Sampling Technique

The study population comprised patients with T2DM receiving oral antidiabetic medication, aged 25–65 years, and followed at the National Institute of Nutrition and Food Technology in Tunis, Tunisia. As previously mentioned, participants were divided into two groups based on diabetes duration: rT2DM with DM history of ≤5 years and cT2DM with a history of >5 years. This 5-year cut-off has been widely used in previous studies assessing complications, metabolic control, and quality of life in patients with T2DM [37,38,39]. Non-inclusion criteria encompassed pregnancy, current smoking status, alcohol consumption, chronic kidney disease, liver disease, and dental diseases, as these conditions could independently affect taste perception.

Systematic sampling was implemented on consultation days with a sampling interval calculated using the formula k = N/n, where k represents the sampling interval, N represents the total number of patients on the consultation day (estimated at 50 patients), and n represents the number of patients to be surveyed per day (set at 5). Therefore, the sampling interval for each consultation day was established at 10 patients (50/5). During consultation days, a number between 1 and 10 was randomly selected, corresponding to the chair number of the first patient included in the waiting room. Subsequently, an interval of 10 was applied until five patients were included per consultation day. The recruitment period lasted 3 months, from April to June 2021.

### 2.4. Data Collection

#### 2.4.1. Anthropometric Measurements

Weight was measured to the nearest 0.1 kg using a calibrated electronic scale with participants wearing light clothing and no shoes. Height was measured to the nearest 0.1 cm using a wall-mounted stadiometer, with participants standing upright, heels together. Body mass index (BMI) was calculated using the standard formula: weight (kg) divided by height squared (m^2^).

#### 2.4.2. Biological Parameters

Biological analyses were performed on blood samples obtained after eight hours of overnight fasting and before conducting the fatty acid orosensory test. The analyses included glycemia (mmol/L), glycated hemoglobin (HbA1c, %), triglycerides (mmol/L), total cholesterol (TC, mmol/L), and high-density lipoprotein cholesterol (HDL-C, mmol/L). Low-density lipoprotein cholesterol (LDL-C, mmol/L) was calculated using the Friedewald formula: LDL-C = TC-HDL-C-(triglycerides/2.2) [40]. Enzymatic methods were employed to determine the concentrations of TC and triglycerides. Glycemia, LDL-C, and HDL-C levels were measured using a UniCel DxC 800 Synchron Clinical Systems analyzer (Beckman Coulter, Inc., Brea, CA, USA). HbA1c concentrations were determined using column chromatography methodology. All laboratory tests were conducted at the National Institute of Nutrition in Tunis, Tunisia, following standardized protocols.

#### 2.4.3. Orosensory Detection of Two Taste Qualities: Fat and Sweet

Taste assessment sessions were conducted to evaluate orosensory detection thresholds for both fat and sweet taste modalities. For fat taste evaluation, linoleic acid was employed as the representative fatty acid stimulus. For sweet taste evaluation, sucrose was used as the prototypical sweet compound. Before data collection, a structured familiarization session was conducted to ensure the reliability of the sensory measurements [41]. All taste tests were conducted under standardized conditions, with identical stimulus preparation, concentration ranges, and test instructions for all participants. The taste stimuli were presented using a standardized forced-choice procedure with three alternatives and increasing concentrations, a commonly used psychophysical method designed to ensure consistent identification of taste detection thresholds.

##### Fat Taste Analysis

Eight solutions containing linoleic acid were prepared for each participant at different ascending concentrations: 0.018, 0.18, 0.37, 0.75, 1.5, 3, 6, and 12 mmol/L. Each solution contained water and 5% Arabic gum (*w*/*v*) to ensure proper emulsification. Additionally, a control solution containing water with 5% Arabic gum (*w*/*v*) without linoleic acid was prepared. This control solution was presented to participants to establish baseline perception and confirm the absence of a fatty taste in the vehicle. The detailed protocol has been previously reported [11]. Taste preference tests for dietary lipids were conducted using a “sip and spit” technique combined with a three-alternative forced-choice method to evaluate participants’ perceptions of taste across various concentrations of linoleic acid. Participants tasted the different concentrations in ascending order, from the lowest to the highest, with appropriate intervals between tastings to minimize sensory adaptation.

##### Sweet Taste Analysis

Sucrose and control solutions were prepared up to 48 h before testing to ensure freshness and stability. Sucrose solutions at increasing concentrations (0.01, 0.02, 0.04, 0.08, 0.16, 0.32, 0.64, 1.25, 2.5, and 5 mmol/L) were prepared by serial dilution of a stock solution with a concentration equal to 6.4 × 10^−1^ mmol/L. Sweet taste tests employed a “sip and spit” technique combined with a three-alternative forced-choice (3-AFC) method to evaluate participants’ perceptions of taste across sucrose concentrations. A control solution containing 15 mL of water was also prepared. Participants tasted the different concentrations in ascending order, from the lowest to the highest.

##### Detection Thresholds

For each taste modality, the detection threshold was operationally defined as the lowest concentration at which participants correctly identified the specific taste quality being evaluated.

### 2.5. Statistical Analysis

The Kolmogorov–Smirnov test was employed to assess the normality of continuous variables. Normally distributed continuous variables were expressed as mean ± standard deviation, while non-normally distributed variables were presented as median (interquartile range). Comparison of continuous variables between groups was performed using an independent-sample t-test for normally distributed data, with effect size calculated as Cohen’s d [42]. Non-normally distributed continuous variables were compared using the Mann–Whitney U test, with effect size calculated using the biserial correlation coefficient [43]. Correlations between continuous variables were tested using Spearman’s rank correlation coefficient. The strength of the correlation coefficient (r) as an absolute value was interpreted as follows: very weak (r: 0.00–0.19), weak (r: 0.20–0.39), moderate (r: 0.40–0.59), strong (r: 0.60–0.79), and very strong (r: 0.80–1.00) [44]. In addition to unadjusted analyses, multivariate linear regression models were used to examine the independent association between DM duration and taste perception thresholds. Taste perception threshold was considered the dependent variable, while age, BMI, sex/gender, and DM group (recent or chronic) were included as covariates. Regression coefficients (β), 95% confidence intervals (CIs), and *p*-values are presented. All statistical analyses were performed using the open-source R software (version 4.5.1; R Core Team, R Foundation for Statistical Computing, Vienna, Austria), with statistical significance set at *p* < 0.05.

## 3. Results

The study included 67 diabetic patients, comprising 30 with rT2DM and 37 with cT2DM, stratified by duration of DM for subgroup analyses, as previously mentioned. 

As detailed in Table 1, the cT2DM group was significantly older than the rT2DM group (54.2 ± 11.6 vs. 44.3 ± 13.5 years, *p* = 0.002) and had a higher BMI (28.5 ± 5.5 vs. 26.4 ± 4.5 kg/m^2^), although this difference did not reach statistical significance (*p* = 0.099). 

Furthermore, the cT2DM group had a significantly longer duration of DM (median 18 [IQR: 10–20] vs. 3 [3,4] years, *p* < 0.001) and a different sex/gender distribution, with a lower proportion of males (18.9% vs. 46.7%, *p* = 0.015). No significant differences were observed between the two groups in terms of weight, treatment modality, prevalence of chronic complications, or any metabolic parameter, including glycated hemoglobin, glycemia, triglycerides, TC, HDL-C, and LDL-C (all *p* > 0.05) (Table 1). Oral antidiabetic agents used in our study included metformin.

### 3.1. Orosensory Detection Threshold for Fat Taste

**Figure 1:**
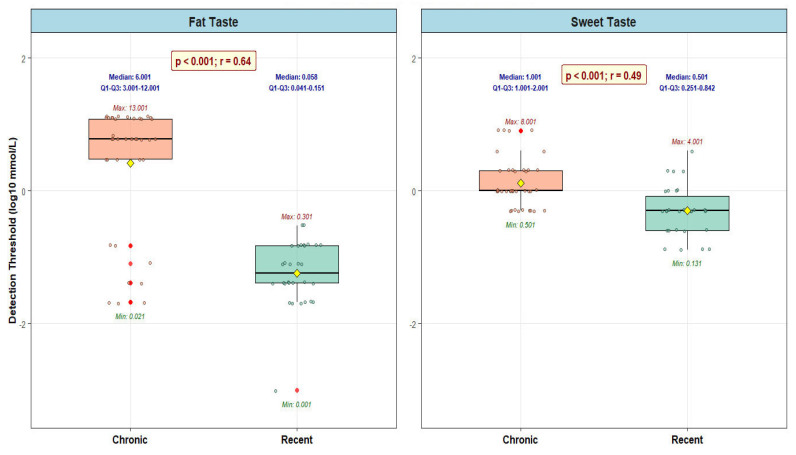
Comparison of Taste Detection Thresholds.

Figure 1 represents the comparison of linoleic acid detection thresholds between rT2DM and cT2DM. Patients with cT2DM demonstrated significantly elevated orosensory detection thresholds for linoleic acid compared to those with rT2DM. The median detection threshold was 6.000 mmol/L in the cT2DM group versus 0.056 mmol/L in the rT2DM group (*p* < 0.001). This represented a 107-fold increase in the concentration required for taste detection in patients with chronic disease, indicating substantially reduced sensitivity to fat taste.

### 3.2. Orosensory Detection Threshold for Sweet Taste

Figure 1 also presents a comparison of sucrose detection thresholds between the rT2DM and cT2DM groups. Similarly, patients with cT2DM exhibited significantly higher orosensory sucrose detection thresholds than those with rT2DM. The median detection threshold was 1.0 mmol/L in the cT2DM group, compared with 0.5 mmol/L in the rT2DM group (*p* < 0.001), representing a 2-fold increase in threshold. This finding indicated that cT2DM patients required twice the sucrose concentration to detect sweet taste as rT2DM patients.

### 3.3. Association Between Diabetes Duration vs. Taste Perception and Metabolic Parameters

**Figure 2:**
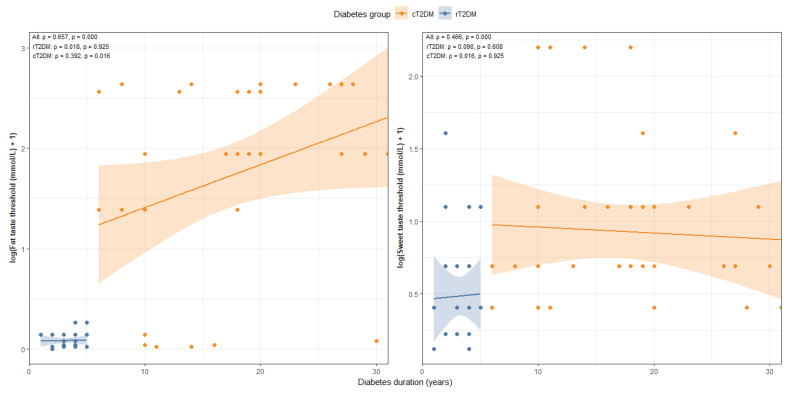
Association Between Diabetes Duration and Taste Perception.

The analysis revealed a strong positive association between diabetes duration and taste perception thresholds in the overall cohort. Significant correlations were observed for both fat taste (r = 0.657, *p* < 0.001) and sweet taste (r = 0.466, *p* < 0.001). At the subgroup level, no significant associations were found in the rT2DM group (fat taste: r = 0.018, *p* = 0.925; sweet taste: r = 0.098, *p* = 0.608). In contrast, within the cT2DM group, the correlation remained significant for fat taste (r = 0.392, *p* = 0.016), while sweet taste showed no significant relationship (r = 0.016, *p* = 0.925) (Figure 2).

**Figure 3:**
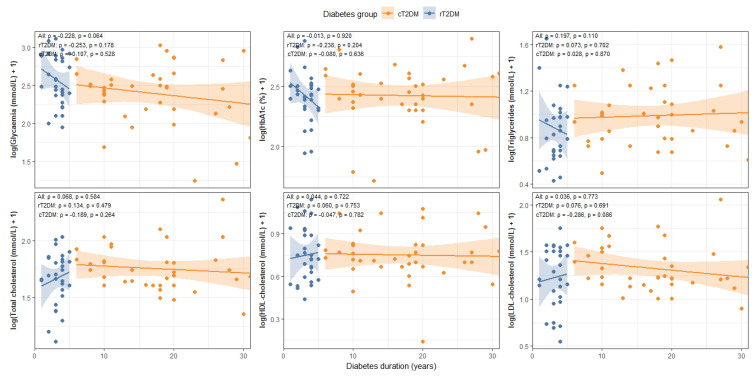
Association Between Diabetes Duration and Metabolic Markers.

Regarding metabolic parameters, diabetes duration showed no significant correlation with HbA1c (r = −0.013, *p* = 0.920), TC (r = 0.068, *p* = 0.584), HDL (r = 0.044, *p* = 0.722), triglycerides (r = 0.197, *p* = 0.110), or LDL in the overall cohort (r = 0.036, *p* = 0.773). A weak, non-significant trend was observed with fasting glycemia (r = −0.228, *p* = 0.064). When analyzed by group, no significant correlations were found in the rT2DM group, whereas in the cT2DM group, a modest but non-significant trend was noted for LDL (r = −0.286, *p* = 0.086) (Figure 3).

### 3.4. Association Between Taste Perception vs. Metabolic Markers

**Figure 4:**
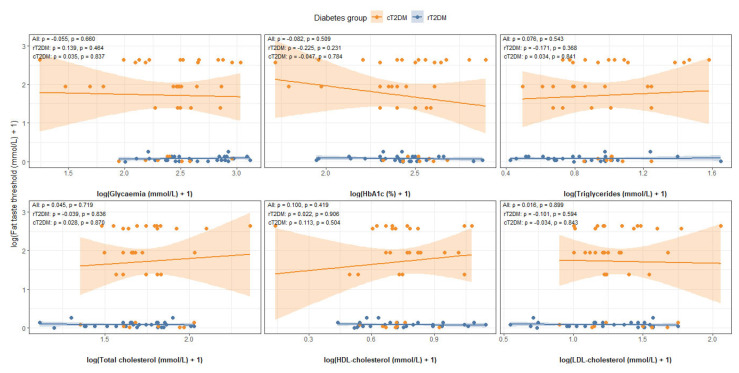
Associations between perception thresholds for fat taste and metabolic markers.

**Figure 5:**
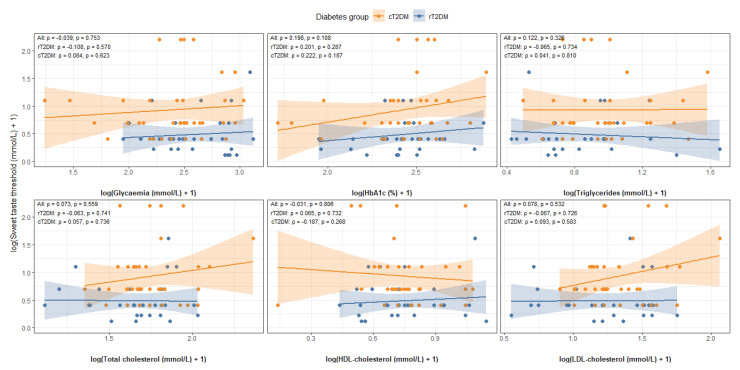
Associations between perception threshold for sweet taste and metabolic markers.

Across the entire cohort, no significant associations were found between taste perception and metabolic parameters. Fat taste sensitivity showed no correlation with fasting glycemia (r = −0.055, *p* = 0.660), HbA1c (r = −0.082, *p* = 0.509), triglycerides (r = 0.076, *p* = 0.543), TC (r = 0.045, *p* = 0.719), HDL (r = 0.100, *p* = 0.419), or LDL (r = 0.016, *p* = 0.899). Similarly, sweet taste sensitivity was not associated with fasting glycaemia (r = 0.039, *p* = 0.753), HbA1c (r = 0.198, *p* = 0.108), triglycerides (r = 0.122, *p* = 0.326), TC (r = 0.073, *p* = 0.559), HDL (r = 0.031, *p* = 0.806), or LDL (r = 0.078, *p* = 0.532) (Figure 4 and Figure 5).

In the rT2DM group, correlations for fat taste ranged from −0.225 to 0.139, with *p*-values between 0.231 and 0.906, while correlations for sweet taste ranged from −0.063 to 0.201, with *p*-values from 0.287 to 0.741—all indicating weak and non-significant relationships.

In the cT2DM group, correlations for fat taste ranged from −0.047 to 0.113 (*p* = 0.504 to 0.870), and correlations for sweet taste ranged from −0.187 to 0.222 (*p* = 0.187 to 0.810), again showing consistently weak and non-significant associations (Figure 4 and Figure 5).

### 3.5. Multivariable Analysis of Taste Perception

In multivariate linear regression analyses, the duration of diabetes remained independently associated with fat taste perception thresholds after adjustment for age, BMI, and sex/gender. More in detail, diabetes group (cT2DM vs. rT2DM) was significantly associated with both fat (β = 1.08, 95% CI: 0.48–1.67; *p* < 0.001) and sweet (β = 0.47, 95% CI: 0.08–0.86; *p* = 0.018) taste perception thresholds, whereas age, BMI, and sex/gender were not significant predictors in the adjusted models (Table 2).

## 4. Discussion

The main finding of this pilot study, which compared two groups of diabetic patients stratified by disease duration, is that patients with cT2DM (duration > 5 years) had significantly higher detection thresholds for the tastes of fat and sweet than those with rT2DM (duration ≤ 5 years). The two groups were otherwise similar with respect to metabolic parameters and diabetes-related complications. These group-level differences suggest a decrease in taste sensitivity with increasing disease chronicity. Unadjusted analyses revealed a positive association between diabetes duration and taste detection thresholds, confirming this observation. Importantly, after adjusting for age, BMI, and sex, diabetes duration remained independently associated with the perception of fatty and sweet taste.

### 4.1. Impaired Fat Taste Detection in Chronic Diabetes

The present investigation demonstrated that patients with cT2DM exhibited a 107-fold increase in the linoleic acid detection threshold compared with those with recent diabetes, indicating a profound impairment in fat taste perception. Taste sensitivity is triggered when the concentration of a stimulus reaches a threshold that activates specific taste receptors, generating action potentials in afferent gustatory nerve fibers sufficient to elicit conscious taste perception [45]. Among the chemoreceptor proteins responsible for taste perception, CD36 (also called fatty acid translocase) plays a central role in the perception of fat taste [5,46]. This substantial elevation in fat taste threshold observed in patients with cT2DM may reflect multiple pathophysiological mechanisms. Previous research has documented that a longer duration of diabetes is associated with microvascular complications affecting small blood vessels throughout the body [25]. Specifically, microangiopathy can alter the density and structure of blood vessels at the tip of the tongue, potentially compromising the nutritional supply to taste buds and affecting their functional integrity [25]. Additionally, the chronic hyperglycemic environment characteristic of prolonged diabetes may lead to glycation of taste receptor proteins, potentially altering their binding affinity for fatty acid ligands [26,27]. The clinical significance of impaired fat taste detection extends beyond sensory function, as individuals with reduced fat taste sensitivity may unconsciously increase their consumption of lipid-rich foods to achieve satisfactory palatability [11,19]. This compensatory behavior could contribute to excessive caloric intake, weight gain, and deterioration of metabolic control, creating a vicious cycle that exacerbates the diabetic condition. Furthermore, the relationship between fat taste sensitivity and metabolic parameters suggests that restoring or enhancing fat taste perception could represent a novel therapeutic target for improving dietary adherence and metabolic outcomes in patients with diabetes [47,48].

### 4.2. Diminished Sweet Taste Sensitivity with Disease Duration

The 2-fold increase in the sucrose detection threshold observed in cT2DM patients compared with rT2DM patients is consistent with previous investigations reporting altered sweet taste perception in T2DM [9,20,21]. A systematic review examining sweet taste function in adults with T2DM supported these findings, demonstrating reduced sensitivity to sweet stimuli in patients with T2DM compared with healthy controls [23]. This observation is consistent with earlier studies that identified diminished sweet taste detection as a factor contributing to heightened sugar preferences and potentially contributing to the onset or progression of diabetes [28,30]. However, some investigations have reported contrasting findings: De Carli et al. observed greater taste sensitivity in uncomplicated T2DM patients [9], suggesting that the presence of complications and disease severity may influence the relationship between diabetes and taste perception. In advanced stages of T2DM, patients may experience further deterioration or even complete loss of their ability to detect sucrose and fat, a condition known as diabetic hypogeusia [31]. This reduced taste sensitivity has been associated with several pathophysiological mechanisms, including microangiopathy, olfactory impairments that can influence overall flavor perception, peripheral neuropathy affecting the trigeminal and glossopharyngeal nerves, and lingual nerve damage [25,31,49,50]. The observed reduction in sweet taste sensitivity may have important clinical implications, as diminished perception of sweetness could potentially lead patients to add more sugar to foods and beverages to achieve desired taste satisfaction [22,51]. This behavioral adaptation may be associated with increased sugar consumption, potentially contributing to poorer glycemic control and accelerated progression of diabetic complications.

### 4.3. Mechanistic Considerations and Clinical Implications

Several interconnected mechanisms have been proposed in the existing body of scholarly literature to potentially explain the alterations in taste perception among individuals with T2DM. These include microangiopathy affecting tongue vasculature [25], reduced glucagon-like peptide-1 signaling, which modulates typical taste sensitivity [29], disordered control of sweet taste intestinal receptors [28], inhibition of gustatory response to sweet compounds induced by hyperleptinemia [30], and diabetic neuropathy affecting both peripheral and autonomic nervous system components [31,49].

Fournel et al. reported a significant relationship between peripheral neuropathy, autonomic neuropathy, and hypogeusia in long-term diabetic patients [28]. Patients with T2DM who have suboptimal glycemic control face heightened risk for complications, including periodontitis and sensory neuropathy, both of which can disrupt taste perception [52]. Oral lesions and periodontitis can cause discomfort during the chewing and swallowing processes [52]. Furthermore, cranial neuropathy associated with diabetes can alter neural pathways, potentially altering how taste signals are perceived and processed [53]. The autonomic nervous system can also be affected, leading to imbalanced salivary production that affects chewing, swallowing, and the dissolution of taste compounds [53]. The consequences of these taste disturbances extend to dietary habits, particularly in sweet taste perception, which may be associated with increased preference for sugar-rich foods and poor metabolic control [22,51]. Research indicates that the signaling mechanisms responsible for sweet taste detection in the oral cavity also function within the gastrointestinal system and influence satiety signals [4,54]. Therefore, impaired taste perception may have cascading effects on multiple levels of metabolic regulation.

### 4.4. Limitations

The current investigation has several limitations that should be acknowledged. First, we did not assess the relationship between taste perception and actual dietary intake patterns, which can independently influence the threshold of orosensory perception regardless of diabetes duration [24]. Second, we did not include non-diabetic control subjects in comparison with diabetic individuals, which limits our ability to determine the absolute degree of impairment relative to healthy populations. The cross-sectional design precludes establishing causal relationships and temporal sequences, preventing us from definitively determining whether reduced taste sensitivity is a cause or a consequence of prolonged diabetes. The relatively small sample size, particularly after stratification into recent and chronic diabetes subgroups, may have reduced statistical power and contributed to variability in the subgroup and correlation analyses, particularly for the perception of sweet taste, thereby limiting the generalizability of the results. Moreover, potential confounding factors, including specific medication regimens, oral health status, periodontal disease severity, gustatory sweating, and detailed dietary habits, were not fully controlled and could have influenced taste perception measurements. The study did not evaluate the presence or severity of diabetic complications such as neuropathy, retinopathy, or nephropathy, which may be important mediators of the relationship between diabetes duration and taste impairment. Additionally, genetic polymorphisms in taste receptor genes, which are known to influence taste sensitivity, were not assessed in this population. Furthermore, no formal assessment of intra- or inter-individual variability (e.g., test–retest reliability) was conducted. Although standardized procedures and a prior familiarization session were implemented to reduce random measurement errors, the lack of repeated measurements may limit the evaluation of the robustness of the measures at the individual level. These limitations may have affected the robustness and validity of our results and should be considered in future research.

Further studies, including longitudinal follow-up, objective assessment of diabetic complications, repeated taste measurements, and detailed characterization of diet and oral health, are needed to elucidate the mechanisms underlying altered taste perception in patients with long-standing T2DM. Although multivariate analyses were performed to control for the main confounding factors in this study, residual bias cannot be completely ruled out.

## 5. Conclusions

Our study demonstrated a significant association between T2DM duration and alterations in taste sensitivity, particularly for sweet and fatty taste modalities. As disease duration progresses from recent to chronic stages, we observed substantial increases in detection thresholds for both sucrose and linoleic acid, with cT2DM patients requiring 2-fold higher sucrose concentrations and 107-fold higher linoleic acid concentrations to detect these tastes than rT2DM patients. These findings suggest reduced chemosensory sensitivity rather than complete loss of taste perception, supporting the hypothesis that prolonged metabolic dysregulation and associated microvascular and neurological complications may progressively alter sensory processing pathways involved in taste perception. The clinical implications of these observations may be relevant, as impaired taste sensitivity could be associated with altered dietary preferences, increased consumption of sugar-rich, fat-dense foods to compensate for reduced hedonic satisfaction, and, consequently, with compromised metabolic control and accelerated disease progression. These findings underscore the importance of incorporating chemosensory function into comprehensive diabetes management and patient counseling. Healthcare providers should be aware that patients with longer disease duration may experience diminished taste sensitivity, which could interfere with adherence to dietary recommendations and affect food choices. Our findings provide preliminary evidence from a MENA population supporting a link between T2DM duration and taste alterations, contributing to the limited data available from this geographical region. However, future longitudinal studies with larger, more diverse cohorts are needed to confirm these cross-sectional associations, establish temporal relationships, and clarify the mechanisms by which diabetes affect chemosensory function. Finally, as previously mentioned, investigations incorporating assessments of diabetic complications, genetic factors influencing taste sensitivity, and detailed dietary intake patterns would provide a more comprehensive understanding of this phenomenon.

## Figures and Tables

**Table 1:** Characteristics of patients with recent (rT2DM) and chronic (cT2DM) diabetes mellitus.

Parameters	Unit or Category	rT2DM (*n* = 30)	cT2DM (*n* = 37)	*p*-Value	Effect Size [95%CI]
Sex/gender	Male	14 (46.7)	7 (18.9)	0.015	0.30
Age	Years	44.3 ± 13.5	54.2 ± 11.6	0.002	0.79 [95%CI 0.29–1.29]
Weight	kg	73.1 ± 12.2	73.3 ± 16.1	0.956	0.01 [95%CI −0.47 to 0.50]
Body mass index	kg/m^2^	26.4 ± 4.5	28.5 ± 5.5	0.099	0.41 [95%CI −0.08 to 0.90]
Duration of diabetes	years	3 (3–4)	18 (10–20)	<0.001	1.00
Treatment of T2DM	Diet	2 (6.7)	6 (16.2)	0.467	0.16
	OAA	8 (26.7)	7 (18.9)		
	Insulin	20 (66.7)	24 (64.9)		
Chronic complications	No	26 (86.7)	25 (67.6)	0.211	0.23
	Microangiopathy	3 (10.0)	8 (21.6)		
	Macroangiopathy	1 (3.3)	4 (10.8)		
HbA1c	%	10.4 ± 2.3	10.6 ± 2.6	0.804	0.06 [95%CI −0.42 to 0.54]
Glycemia	mmol/L	12.8 ± 4.5	10.8 ± 4.2	0.067	−0.46 [95%CI −0.94 to 0.03]
Triglycerides	mmol/L	1.52 ± 0.84	1.77 ± 0.74	0.204	0.32 [95%CI −0.17 to 0.80]
Total Cholesterol	mmol/L	4.44 ± 1.16	4.90 ± 1.28	0.135	0.37 [95%CI −0.12 to 0.86]
HDL-C	mmol/L	1.15 ± 0.43	1.15 ± 0.37	0.972	−0.01 [95%CI −0.49 to 0.47]
LDL-C	mmol/L	2.58 ± 1.06	2.89 ± 1.10	0.242	0.29 [95%CI −0.20 to 0.77]

Abbreviations: HbA1c (glycated hemoglobin); HDL-C (high-density lipoprotein cholesterol); LDL-C (low-density lipoprotein cholesterol); OAA: oral anti-diabetic agent. Categorical data were numbers (%). Quantitative data were reported as mean ± standard deviation, except for DM duration, which was reported as median (interquartile range). *p*-values (2-sided chi-squared or Student test, parametric or non-parametric): rT2DM vs. cT2DM.

**Table 2:** Multivariable linear regression analysis of determinants of taste perception thresholds.

Outcome	Predictor	β (95% CI)	*p*-Value
Fat taste	Age (years)	−0.003 (−0.018–0.011)	0.643
	BMI (kg/m^2^)	−0.004 (−0.039–0.031)	0.829
	Sex	0.049 (−0.360–0.457)	0.813
	Diabetes group	1.08 (0.48–1.67)	<0.001
Sweet taste	Age (years)	0.007 (−0.002–0.017)	0.141
	BMI (kg/m^2^)	−0.003 (−0.026–0.020)	0.812
	gender	−0.133 (−0.400–0.134)	0.324
	Diabetes group	0.47 (0.08–0.86)	0.018

Note: β coefficients were obtained from multivariable linear regression models with log-transformed taste detection thresholds as dependent variables. Models were adjusted for age, BMI (body mass index), sex/gender, and diabetes group (rT2DM vs. cT2DM).

## Data Availability

The data supporting the findings of this study are available from the corresponding authors upon reasonable request.

## References

[1] Piemonte L. (2019). Diabetes Atlas Reports 463 Million with Diabetes.

[2] Barragán R., Coltell O., Portolés O., Asensio E.M., Sorlí J.V., Ortega-Azorín C., González J.I., Sáiz C., Fernández-Carrión R., Ordovas J.M. (2018). Bitter, Sweet, Salty, Sour and Umami Taste Perception Decreases with Age: Sex-Specific Analysis, Modulation by Genetic Variants and Taste-Preference Associations in 18 to 80 Year-Old Subjects. Nutrients.

[3] Mbarki S., Ben Abdelaziz A., Ben Hassine D., Melki S., Ben Rejeb N., Omezzine A., Bouslama A., Ben Abdelaziz A. (2022). Epidemiology of diabetes mellitus in Tunisia. HSHS 2 study (Hammam Sousse Sahloul Heart Study). Tunis. Med..

[4] Simon S.A., de Araujo I.E., Gutierrez R., Nicolelis M.A.L. (2006). The neural mechanisms of gustation: A distributed processing code. Nat. Rev. Neurosci..

[5] Besnard P., Passilly-Degrace P., Khan N.A. (2016). Taste of Fat: A Sixth Taste Modality?. Physiol. Rev..

[6] Stolbová K., Hahn A., Benes B., Andel M., Treslová L. (1999). Gustometry of diabetes mellitus patients and obese patients. Int. Tinnitus J..

[7] Mwakyoma T., Graham C.A.-M., Hamooya B.M., Muchaili L., Ngosa M., Povia J.P., Pilic L., Masenga S.K. (2025). Gene variations and sweet taste sensitivity in Zambian adults with and without type 2 diabetes mellitus. PLoS ONE.

[8] Keskitalo K., Knaapila A., Kallela M., Palotie A., Wessman M., Sammalisto S., Peltonen L., Tuorila H., Perola M. (2007). Sweet taste preferences are partly genetically determined: Identification of a trait locus on chromosome 161. Am. J. Clin. Nutr..

[9] De Carli L., Gambino R., Lubrano C., Rosato R., Bongiovanni D., Lanfranco F., Broglio F., Ghigo E., Bo S. (2018). Impaired taste sensation in type 2 diabetic patients without chronic complications: A case-control study. J. Endocrinol. Investig..

[10] Gutierrez R., Simon S.A. (2011). Chemosensory processing in the taste—Reward pathway. Flavour Fragr. J..

[11] Karmous I., Plesník J., Khan A.S., Šerý O., Abid A., Mankai A., Aouidet A., Khan N.A. (2018). Orosensory detection of bitter in fat-taster healthy and obese participants: Genetic polymorphism of CD36 and TAS2R38. Clin. Nutr..

[12] Perez-Aguilar J.M., Kang S.-G., Zhang L., Zhou R. (2019). Modeling and Structural Characterization of the Sweet Taste Receptor Heterodimer. ACS Chem. Neurosci..

[13] Belloir C., Brulé M., Tornier L., Neiers F., Briand L. (2021). Biophysical and functional characterization of the human TAS1R2 sweet taste receptor overexpressed in a HEK293S inducible cell line. Sci. Rep..

[14] Batisse C., Bonnet G., Eschevins C., Hennequin M., Nicolas E. (2017). The influence of oral health on patients’ food perception: A systematic review. J. Oral. Rehabil..

[15] Laugerette F., Gaillard D., Passilly-Degrace P., Niot I., Besnard P. (2007). Do we taste fat?. Biochimie.

[16] Mortazavi H., Shafiei S., Sadr S., Safiaghdam H. (2018). Drug-related Dysgeusia: A Systematic Review. Oral. Health Prev. Dent..

[17] Ben Othman R., Karmous I., Aissa F., Ceylan H.I., Zanina Y., Jamoussi H., Bragazzi N.L., Dergaa I. (2025). Reduced Fat Taste Sensitivity and Its Association with Childhood Obesity in Tunisian Children: A Cross-Sectional Study. Nutrients.

[18] Kaufman A., Choo E., Koh A., Dando R. (2018). Inflammation arising from obesity reduces taste bud abundance and inhibits renewal. PLoS Biol..

[19] Noel C.A., Sugrue M., Dando R. (2017). Participants with pharmacologically impaired taste function seek out more intense, higher calorie stimuli. Appetite.

[20] Catamo E., Tornese G., Concas M.P., Gasparini P., Robino A. (2021). Differences in taste and smell perception between type 2 diabetes mellitus patients and healthy controls. Nutr. Metab. Cardiovasc. Dis..

[21] Pugnaloni S., Alia S., Mancini M., Santoro V., Di Paolo A., Rabini R.A., Fiorini R., Sabbatinelli J., Fabri M., Mazzanti L. (2020). A Study on the Relationship between Type 2 Diabetes and Taste Function in Patients with Good Glycemic Control. Nutrients.

[22] Perros P., MacFarlane T.W., Counsell C., Frier B.M. (1996). Altered taste sensation in newly-diagnosed NIDDM. Diabetes Care.

[23] Tan S.-Y., Hack C., Yu C., Rennick I., Ohanian J., Dezan M., Mott N., Manibo R., Tucker R.M. (2023). Alterations in sweet taste function in adults with diabetes mellitus: A systematic review and potential implications. Crit. Rev. Food Sci. Nutr..

[24] Karmous I., Ben Othman R., Dergaa I., Ceylan H.I., Bey C., Dhahbi W., Khan A.S., Jamoussi H., Muntean R.I., Khan N.A. (2025). Sweet and Fat Taste Perception: Impact on Dietary Intake in Diabetic Pregnant Women-A Cross-Sectional Observational Study. Nutrients.

[25] Pavlidis P., Gouveris C., Kekes G., Maurer J. (2014). Changes in electrogustometry thresholds, tongue tip vascularization, density and form of the fungiform papillae in smokers. Eur. Arch. Oto-Rhino-Laryngol..

[26] Young R.L., Sutherland K., Pezos N., Brierley S.M., Horowitz M., Rayner C.K., A Blackshaw L. (2009). Expression of taste molecules in the upper gastrointestinal tract in humans with and without type 2 diabetes. Gut.

[27] Bloomfeld R.S., Graham B.G., Schiffman S.S., Killenberg P.G. (1999). Alterations of chemosensory function in end-stage liver disease. Physiol. Behav..

[28] Fournel A., Marlin A., Abot A., Pasquio C., Cirillo C., Cani P.D., Knauf C. (2016). Glucosensing in the gastrointestinal tract: Impact on glucose metabolism. Am. J. Physiol.-Gastrointest. Liver Physiol..

[29] Martin B., Dotson C.D., Shin Y., Ji S., Drucker D.J., Maudsley S., Munger S.D. (2009). Modulation of taste sensitivity by GLP-1 signaling in taste buds. Ann. N. Y. Acad. Sci..

[30] Loper H.B., La Sala M., Dotson C., Steinle N. (2015). Taste perception, associated hormonal modulation, and nutrient intake. Nutr. Rev..

[31] Le Floch J.P., Le Lievre G., Sadoun J., Perlemuter L., Peynegre R., Hazard J. (1989). Taste impairment and related factors in type I diabetes mellitus. Diabetes Care.

[32] Tesfaye S., Boulton A.J., Dyck P.J., Freeman R., Horowitz M., Kempler P., Lauria G., Malik R.A., Spallone V., Vinik A. (2010). Diabetic neuropathies: Update on definitions, diagnostic criteria, estimation of severity, and treatments. Diabetes Care.

[33] Al-Sari N., Kutuzova S., Suvitaival T., Henriksen P., Pociot F., Rossing P., McCloskey D., Legido-Quigley C. (2022). Precision diagnostic approach to predict 5-year risk for microvascular complications in type 1 diabetes. eBioMedicine.

[34] Hartman-Petrycka M., Knefel G., Lebiedowska A., Nowak M., Błońska-Fajfrowska B. (2022). Taste perception and food preferences in patients with diabetic foot ulcers before and after hyperbaric oxygen therapy. Nutr. Diabetes.

[35] Keith M., Mokbel R., San Emeterio M., Song J., Errett L. (2010). Evaluation of taste sensitivity in patients undergoing coronary artery bypass graft surgery. J. Am. Diet. Assoc..

[36] The World Medical Association (2021). WMA Declaration of Helsinki—Ethical Principles for Medical Research Involving Human Subjects. https://www.wma.net/policies-post/wma-declaration-of-helsinki/.

[37] de Waard E.A.C., Koster A., Melai T., van Geel T.A., Henry R.M.A., Schram M.T., Dagnelie P.C., van der Kallen C.J., Sep S.J.S., Stehouwer C.D.A. (2016). The association between glucose metabolism status, diabetes severity and a history of fractures and recent falls in participants of 50 years and older—The Maastricht Study. Osteoporos. Int..

[38] Herold M., Szasz A.M., Szentmartoni G., Martinek E., Madar-Dank V., Barna A.J., Mohacsi R., Somogyi A., Dank M., Herold Z. (2023). Influence of the duration of type 2 diabetes mellitus on colorectal cancer outcomes. Sci. Rep..

[39] Barua L., Faruque M., Chowdhury H.A., Banik P.C., Ali L. (2021). Health-related quality of life and its predictors among the type 2 diabetes population of Bangladesh: A nation-wide cross-sectional study. J. Diabetes Investig..

[40] Friedewald W.T., Levy R.I., Fredrickson D.S. (1972). Estimation of the concentration of low-density lipoprotein cholesterol in plasma, without use of the preparative ultracentrifuge. Clin. Chem..

[41] Giacalone D., Hedelund P.I. (2016). Rate-all-that-apply (RATA) with semi-trained assessors: An investigation of the method reproducibility at assessor-, attribute- and panel-level. Food Qual. Prefer..

[42] Espirito Santo H., Daniel F. (2017). Calcular E Apresentar Tamanhos Do Efeito EM Trabalhos Científicos (1): As Limitações Do P < 0,05 Na Análise De Diferenças De Médias De Dois Grupos (Calculating and Reporting Effect Sizes on Scientific Papers (1): P < 0.05 Limitations in the Analysis of Mean Differences of Two Groups). Rev. Port. Investig. Comport. Soc..

[43] Gardner M.J., Altman D.G. (1986). Confidence intervals rather than P values: Estimation rather than hypothesis testing. Br. Med. J. (Clin. Res. Ed.).

[44] Campbell M.J., Swinscow T.D.V. (2011). Statistics at Square One.

[45] Pepino M.Y., Mennella J.A. (2007). Effects of cigarette smoking and family history of alcoholism on sweet taste perception and food cravings in women. Alcohol. Clin. Exp. Res..

[46] Kampov-Polevoy A.B., Tsoi M.V., Zvartau E.E., Neznanov N.G., Khalitov E. (2001). Sweet liking and family history of alcoholism in hospitalized alcoholic and non-alcoholic patients. Alcohol Alcohol..

[47] Yu J.H., Shin M.S., Kim D.J., Lee J.R., Yoon S., Kim S.G., Koh E.H., Lee W.J., Park J., Kim M. (2013). Enhanced carbohydrate craving in patients with poorly controlled Type 2 diabetes mellitus. Diabet. Med..

[48] Schwingshackl L., Hoffmann G., Lampousi A.-M., Knüppel S., Iqbal K., Schwedhelm C., Bechthold A., Schlesinger S., Boeing H. (2017). Food groups and risk of type 2 diabetes mellitus: A systematic review and meta-analysis of prospective studies. Eur. J. Epidemiol..

[49] Duda-Sobczak A., Araszkiewicz A., Urbas M., Borucki L., Kulas K., Chudzinski M., Suwalska A., Zozulinska-Ziolkiewicz D. (2017). Impaired olfactory function is related to the presence of neuropathy in adults with type 1 diabetes. Diabetes Vasc. Dis. Res..

[50] Borgnakke W.S., Anderson P.F., Shannon C., Jivanescu A. (2015). Is there a relationship between oral health and diabetic neuropathy?. Curr. Diab Rep..

[51] Low Y.Q., Lacy K., Keast R. (2014). The role of sweet taste in satiation and satiety. Nutrients.

[52] Boulton A.J., Vinik A.I., Arezzo J.C., Bril V., Feldman E.L., Freeman R., Malik R.A., Maser R.E., Sosenko J.M., Ziegler D. (2005). Diabetic neuropathies: A statement by the American Diabetes Association. Diabetes Care.

[53] Ashi H., Campus G., Forslund H.B., Hafiz W., Ahmed N., Lingström P. (2017). The Influence of Sweet Taste Perception on Dietary Intake in Relation to Dental Caries and BMI in Saudi Arabian Schoolchildren. Int. J. Dent..

[54] Shu-Fen C.L., Forde C.G., Tey S.L., Henry C.J. (2018). Taste sensitivities and diet of Chinese and Indians in Singapore. Asia Pac. J. Clin. Nutr..

[55] Dergaa I., Fekih-Romdhane F., Glenn J.M., Fessi M.S., Chamari K., Dhahbi W., Zghibi M., Bragazzi N.L., Ben Aissa M., Guelmami N. (2023). Moving Beyond the Stigma: Understanding and Overcoming the Resistance to the Acceptance and Adoption of Artificial Intelligence Chatbots. New Asian J. Med..

[56] Dergaa I., Ben Saad H., Glenn J.M., Ben Aissa M.M., Taheri M., Swed S., Guelmami N., Chamari K. (2024). A thorough examination of ChatGPT-3.5 potential applications in medical writing: A preliminary study. Medicine.

